# Indents

**Published:** 1917-03

**Authors:** 


### INDENTS

**☞ Brief Articles of Technical or Scientific Value will receive notice and comment. Contributions invited.**

*Fixed Bridges Iniquitous.* Fixed bridgework is fundamentally an iniquity because it does not conform to the fundamental demand of nature. It has no natural prototype. In all the hundreds of thousands of years during which the creative principle has been at work in this world it has never created a human being whose natural teeth were normally united into one fixed mass. Whenever two or more natural teeth, because of some untoward interruption in normal growth and development, were fused into one mass, it became only a question of time as to when the human economy would demand its removal in an unmistakable way.—Dr. Chayes in February *Items of Interest.*

*The Bridge Span.* No bridge restoration should be placed in edentulous space which can not exercise the subjacent or spanned tissues, by means of a properly adjusted dummy or saddle, which should replace the function in that area formerly carried on by the roots of the natural teeth.—Dr. Chayes.

*Limitation of Fixed Bridgework.* Dr. Thornton says he believes that fixed bridgework should be confined to the anterior part of the mouth, because there it can be readily cleaned. I can not follow him here—I believe fixed bridgework should be confined to the dead past, to the heap of all other unreasonable methods of practice which were in vogue and very popular during the Spanish Inquisition, with all its subtle tortures. Fixed bridgework is bad for any space in any mouth under any condition. It is bad because fixation is bad. Fixation induces an inhibition of function, an inhibition of circulation and inhibition of that flow of vital essence upon which life itself depends. If the area is small, the damage is small; but it is there, nevertheless, and I do not want it there.

It is up to the schools. What will they do? Will they continue to teach these methods of bridgework which have crippled untold thousands or will they display the necessary courage and honesty to revise the curriculum and substitute a real safe and sane method based upon the recognition of the primary principle that natural teeth move in function and that artificial restorations must not only never interfere with the movement of pier teeth, but must in themselves simulate this motion as required?—Dr. Chayes, in February *Items of Interest.*

*Sterilization of Instruments.* Instruments that are injured by boiling, but must be rendered aseptic, can be very satisfactorily sterilized in the following solution: Mercuric iodide, 3/8 grain, sodium bicarbonate, 16 grains, water, 4 ounces.

These can be obtained from Parke, Davis Company in tablet form, the socalled Germicidal Discs. This solution has no bad effect upon steel or nickel.—F. W. Fraham, in *Pacific Gazette.*

*Arthritis and Oral Sepsis.* A study of the relation of local dental infections to arthritis has recently been reported by F. L. Morey, Acting Assistant Dental Surgeon U. S. Navy, in the U. S. *Naval Medical Bulletin.*

In a series of six cases of arthritis of long standing, the symptoms subsided immediately or a short time following the eradication of intraoral foci of infection, the supposition being that the mouth streptococcus had been carried by the hematogenic route to the points of activity in the articulations. No attempt is made by the reporter to connect all cases of arthritis with diseased teeth, but on the other hand, he brings forth additional and convincing proof that a large number of cases of arthritis, nephritis and cardiovascular diseases and inflammations in the gastrointestinal tract are of dental etiology.

In order to have a clear understanding of the pathologic phenomena involved in the transportation of bacteria and bacterial toxines from the teeth to remote areas of the body, we venture to suggest to physicians and dentists alike a careful study of the histology and anatomy of the investing tissues of the teeth and of the osseous substance of the jaws. Nothing else will convey a clearer and more definite idea of the reasons why an infection about the roots of the teeth can, under no circumstances, be considered as a strictly localized infection. The blood supply of the peridental membrane and that of the surrounding alveolar structures are not separate and independent vascular areas. The vessels anastomose freely among themselves. An infection located about the apex of a root proceeds until the cancellated substance becomes involved. The cortical layer of bone lining the alveolus soon breaks down as the result of the infectious processes which develop in the periapical space and when this cortical layer disintegrates the infection at once reaches the cancellated bone of the alveolar walls—a continuation of the cancellated spaces in the jaw proper. The involvement of the alveolar walls is in the nature of a septic osteomyelitis. The medullary substance contained in the cancellated spaces becomes the seat of a chronic inflammation in which osteoclasts play an active part. Through osteoclastic action the hard substance of the bone disappears, the cancellated spaces are eaten through and Haversian canals are widened. The contents of the spaces become the seat of a typical chronic inflammation containing large masses of small, round cells. The bone lamellae are successively destroyed and carried away by osteoclasts and in time the inflamed medullary substance breaks down.

The point we wish to emphasize at this time is the one concerning the thickness (or should we say thinness?) of the cortical layer of the alveoli. In some places this cortical layer is less than one-third of a millimeter in thickness, and consequently it is quickly broken down, thereby opening up an abundance of avenues for the absorption of bacteria and bacterial toxines. An acute or chronic apical infection is practically at no time a localized infection. If all chronic infections of roots do not give rise to systemic manifestations, it is not because the bacterin and toxines concerned in the infection are not in all cases beyond the possibilities of absorption, but because individuals are frequently able to ward off successfully the bacterial invasion. In practically all reports on the systemic manifestations of septic foci in the mouth, intraoral foci about the roots of teeth, as well as advanced socalled pyorrheal conditions, are amply discussed. We fail to see why bacterial toxin absorption occurring in conjunction with the milder forms of extraoral foci are so seldom associated with general toxemia. We view the initial steps in gingivitis, viz., the destruction of the epithelial lining of the subgingival space, consequent upon irritation followed by infection, and the destruction of the fixed tissue elements of the underlying connective tissue fibrous mat and their replacement by inflammatory cells—round cell infiltration—as a prolific source for bacteria and bacterial-toxin absorption with all its pathologic manifestations in remote regions of the body. Clinically it has been demonstrated that general toxemia is markedly improved and with continued care completely eradicated, in many cases, following proper surgical and therapeutic treatment of inflamed gingivae. Systemic manifestations of septic foci in the mouth result not only from periapical chronic infections and well established pyorrheal conditions, but more frequently than the casual observer would be inclined to recognize following upon chronic forms of marginal gingivitis. Inflamed gingivae are a menace to the general health even long before the peridental membrane and alveolar process have become affected. Not only the intraoral foci should be eradicated. The extraoral foci are at least as important as the intraoral, if not more so, because of the extent of tissue surface involved and of the systemic complications to which they give rise.—Editorial in *Pacific Dental Gazette.*

*Saving Pulpless Teeth.* What is the cost of a failure to save in a healthy condition a pulpless tooth? The estimate of the past has been only a tooth; but the estimate of the present day may be years of suffering, and possibly a human life.—Dr. W. A. Price.

*Filling Root Canals Aseptically.* Some dentists have the idea that it would be a difficult thing to treat a root canal aseptically. It is not so. It is not as complicated a proposition as one might think. In the first place, I think some of the things which have been suggested in that connection are impractical. I think it is impractical for a dentist to do these operations with sterile hands. It is impractical under the conditions which we must face today in operating. It is not practical for a dentist to have his hands sterile and keep them so during pulp canal treatment, nor is it as essential as in the case of a surgeon. The idea is impractical because for it to be practical the dentist must have surroundings and equipment of a surgical operating room. Of course, it is not impossible, but it is impractical today. I think it better to have an aseptic technic for pulp canal treatment which does not require that our hands be absolutely sterile. This can be simply done. We must first have the rubber dam, because we must have the field of operation sterile or else there is no need to have anything sterile. Mention was made by Dr. Coolidge that after the rubber dam is in place, the teeth included should be rendered aseptic by the use of some antiseptic solution. For the instruments that are to go into the canal, except those which carry dressings or cotton upon them, all we need is two dishes, one with phenol and another with alcohol, and if our instruments are clean and immersed in phenol and then in alcohol, they are safe. You can immerse broaches, plugger points with guttapercha cones attached, and burs, without taking the burs out of the handpiece, in these solutions. The dressings that go in the canal, if the hands are not sterile, must be sterilized after the cotton is wrapped on the broaches. It is a simple thing for the operator to have a number of broaches with metal handles so that heat will not hurt them and a dry oven in which these may be sterilized. The heat for such an oven may be obtained from an electric lamp or a small heating element.—Dr. A. D. Black, in *Dental Review.*

*Guttapercha as a Root Canal Filling.* In the light of our knowledge today of focal infection, it would be fortunate indeed if it could be correctly stated that all of the materials used for filling root canals in the past, except guttapercha, have been discarded. All, with that one exception, have been tried and found wanting. The radiograph is proof of the correctness of this statement. In my opinion, which is also shared by men like Black, Rhein, Callahan, and others, guttapercha, modified and used in some manner as to best meet the needs of the individual operator, is the only material at our command upon which reliance may be placed. From experiments made, Price is even skeptical regarding guttapercha on account of its inherent tendency to shrink, but as against laboratory experiments the records of thousands of clinical cases prove conclusively that this material, properly used, answers our every purpose for filling the canals of pulpless teeth. This being true, the proposition resolves itself into the problem of determining the best method of modifying and using the material. We may modify the properties of guttapercha by incorporating within its substance certain drugs, and drugs are added to root filling materials for two purposes: one a pharmacomechanical reason, the other on a therapeutic basis. In the case of guttapercha it is admissible to add to and use in connection with the material such drugs as chloroform and eucalyptol for pharmacal and mechanical purposes. In the opinion of Callahan, even rosin may be added on this basis. Chloroform and eucalyptol will soften the material which permits its adaptation, by proper manipulation and with heat and instruments, to every irregularity in the canal which has been cleaned and opened by previous treatment. Thus the latter is completely filled with nonabsorbable and, if properly manipulated, I believe, nonshrinkable material. Whether or not we are justified in adding drugs to root filling materials today for therapeutic purposes is a debatable question.—Dr. J. P. Buckley, in *Dental Review*.

*Bad Advice on Extracting Teeth.* Some years ago—I do not remember how long, but it must have been twenty or more years—a family living in this city were patients of mine, and later when I stopped filling teeth they went elsewhere. A woman, now about thirty-five years of age, who was a girl at the time when I first knew her, came to see me within the last year and stated that she was in great despair and wanted my advice. I said to her, "What is the matter?" She replied, "I have been advised to have my upper teeth taken out." I asked her for what purpose, and she replied, "Because I have neuritis and my physician says it is caused directly by these teeth; that there is no other recourse, and I must have my teeth extracted. It is imperative." I said, "Are you going to have it done?" She replied, "I do not know." I looked at her teeth and found that her incisors were all good; they were not pulpless; the pulps were alive; the cuspids were normal. She had filled roots, I think, in bicuspids on the right side and one molar. The second molar had a dead pulp in it. I had skiagraphs made and found that the tooth roots were well filled. There was a little darkened area about one, but it was not sensitive to pressure. There was no indication of trouble. Then I found a root on the opposite side which had a darkened area about it and I told her she had better have that out, and not any other. She had that taken out, and still the pain continued. After another skiagraph was made, taking in the two bicuspid teeth on the right side above and two molars, I observed something that I did not notice the first time. I had the plate at a meeting of this society one night and handed it to the members and they did not see what I found until it was pointed out. In the second molar there was a pulp mass of secondary dentine, a pulp nodule in the chamber. The tooth root above referred to was taken out under the most careful precautions, the surface all around it was sterilized, the tooth root was placed in sterile gauze. Some of the contents at the end were removed with a fine spoon, and this was placed in three sterile tubes; these tubes were sent to three different laboratories, no one laboratory knowing that a tube had been sent to other laboratories. They were examined and pronounced by each one of these pathologists to be sterile. The cause of the trouble in that girl's case was a pulp nodule, and when the chamber of the tooth was opened by the dentist and the pulp destroyed and the pulp nodule removed, that was the end of her pain or neuritis. She has not had any pain since.

Now then, supposing that girl had taken the advice of the physician and had her upper teeth removed, together with the others, that physician would have said, "Didn't I tell you so? You are now free from pain; your teeth were the cause of the pain;" and the girl, assuming he was right, would not have known any better.

In this case the nerve irritation set up by the pulp nodule is proven by the fact that the nodule was taken out and that ended her trouble.—Dr. T. W. Brophy, in *Dental Review.*

*Is Clean Bridgework Practicable?* The patient who has suffered the misfortune to put him in the position of a candidate for a bridge of fourteen teeth, or one of any other length for that matter, should be willing to pay the cost, not only in money for the bridge, but in time and energy sufficient for the use of a toothbrush with paste or powder—a toothpick and floss silk and to rinse his mouth well with water with or without an antiseptic before and after the operation. And I aver that if he will do this with a reasonable amount of intelligence to a properly constructed fixed bridge, of whatever length, he may keep it reasonably clean—as clean as he may keep his natural teeth under normal conditions and cleaner than he can probably keep his natural teeth in many cases where pyorrhea has done its work of destruction in exposing the bifurcations of the molars. If he will not do this, and has not somebody to do it for him, he ought to go dirty and pay the consequences, and I shall shed no tears over him.

I do not want to be understood as being lax in matters of mouth sanitation, because it is not true. But there are some things that are up to the patient, and when I bear my responsibilities I have a right to expect that he will bear his. If I warn a patient of impending danger to a carious tooth, and he says, "Yes, some time, but I'm very busy just now," and comes again after long neglect with it thumping his head off, I give him relief in my best possible manner, but I do not weep over him.

I should like to ask in rebuttal if it is possible to cleanse a plate of any material, constructed in any form, and with any number of teeth, from one to fourteen? The answer, of course, is "Yes." Then I should like to ask how many plates any of us ever saw that would pass inspection as being properly cleansed, and if your experience coincides with mine, even in a practice of great respectability, you will be compelled to answer, "Very few."—Dr. Burgess, in February *Items of Interest.*

*Sacredness of a Vital Tooth.* In the presence of a normal tooth I feel that I stand upon holy ground. The human tooth is a wonderful creation of nature, and we may be sure she has left out of it nothing that was necessary nor put into it anything that is superfluous, and we should enter its sacred precincts with a feeling akin to reverence. In the present state of our knowledge he is a bold and intrepid operator, or a criminally careless one, who will enter a pulp chamber on any mission short of absolute necessity, or else we must put him in that class of benighted mortals of whom it is said that they "rush in where angels fear to tread."

I know of the pathological changes it is feared any invasion of the tooth tissues may bring to the pulp and the ultimate consequences, and I know too, the old argument that "an ounce of prevention is worth a pound of cure." But is this argument applicable to the case in hand? When we have removed the pulp are we sure we have prevented anything? Have we really removed the danger point? Have we not rather merely shifted it from the inside of the tooth where there is a much greater chance that nature will take care of it to the periapical area, thereby increasing manyfold the ultimate danger not only to the tooth itself, but of systemic invasion?—Dr. J. K. Burgess, in February *Items of Interest.*

*Failure of Bridge-work.* "What constitutes a failure?" Men speak of failures of bridgework of all kinds; but what is the standard of failure—the time the bridge lasts? The amount of service given while it lasts, or when the roots and bridges are extracted and discarded?

If these are the definitions of failure, I think, gentlemen, none of us should be censured. Nature made a failure in not permitting the teeth to last long enough, and the condition presented thereby is a failure of nature, and when we take it up at that point we are simply trying to overcome a failure of nature; and if we make a second failure we are no worse off.

A failure in bridgework has not been described as anything more than loss of the teeth.

I have done bridgework probably as long as anyone in the room, and I have seen a great many "failures" because I have at home boxes full of bridges, with teeth on them, that have been extracted. But I do not feel they were failures, because they have done their duty. I hardly think there is a patient but has expressed satisfaction at the work done for them.—Dr. H. W. Northrop in February *Items of Interest.*

*Mobility of Bridge Teeth.* Natural teeth have individual movement of limited range. But if several pier teeth be bound together in a bridge, any motion of an individual tooth must result either in loosening of the bridge from one or more of the pier teeth, or in loosening one or more of the pier roots from the alveolar process. Which is likely to give way first, the cementing medium, or the union between the root and its alveolar connections?—Dr. Thornton, in *Items of Interest.*

*The Fixed Bridge.* In my advocacy of the fixed bridge I do not want to be considered as one who has entrenched himself in a position which he is willing to defend against the invasion of light and truth, but as one who, to the present time, has found more of truth and less of error in that position than in any other. —Dr. Burgess, in *Items of Interest.*

*They Knew Him.* The physician was a guest at a social affair, and at dinner was placed beside an elderly lady whom he had not previously met. Almost at once the lady, who was inclined to garrulity, began to talk.

"By the way, doctor," she smilingly remarked, "ought I to call you 'doctor' or 'professor'?"

"You may call me what you please, madam," was the physician's quick reply. "I am frank enough to admit, however, that some of my friends call me an old fool."

"I see, doctor," smilingly replied the lady, "but, of course, they must be people who know you intimately."

*Modified Crib Clasp.* Dr. D. M. Shaw describes in the *British Dental Record,* a modification of what is known in this country as the Jackson Crib, which gives a more secure clinch to this form of clasp. The modification consists of forming the clasp so that two small projections of wire from the buccal perpendicular portion pass into the buccal embrasure and can be adjusted to any tension desired of the mesial and distal surfaces of the crown to which it is adjusted. These projections may be soldered to the perpendicular portion of the clasp, or better the part of the clasp which fits the buccal surface of the tooth from mesial to distal at the gingival margin is made as a separate piece, and as it turns occlusally at the mesiobuccal and distobuccal angles it makes a second bend which carries its ends into the interproximal space about one-third of the distance from the cervical to the occlusal surface of the crown. The rest of the clasp is completed in two pieces of wire with one end soldered to the cervical part at the angle made by the turn into the interproximal space, and this wire is then bent over the crown to the lingual side of the tooth, through the space formed by the marginal ridges of the approximating teeth. The author makes an interesting statement that he published an account several years ago of a ''crib'' clasp which was illustrated in a book published in London more than forty years ago. It would be interesting to see this book.

*The Ideal Dentist.* The practitioner whom I believe to be ideal is the one who in addition to having a high moral sense so far as professional obligation is concerned, combines with this a keenness of business acument sufficient to conserve his material interests and make him independent in his old age. And this combination is one not at all impossible of achievement by men of even average ability if they are willing to conscientiously devote their energies to a consummation of the task.

It is the average man we most wish to reach. The exceptional man needs no direction at our hands, the hopelessly indifferent or incompetent—well, I like to think that there is none of that class in dentistry, but if we have any we can not reach them by argument or persuasion. The only thing we can do is to pray for them, and I greatly doubt that even that will save them. At least let us pray.—C. N. Johnson, in *Journal of N. D. A.*

*Amputations.* The force of the collision throwed the two men who were in the rear seat against the front seats with such force that both of them were broken off at the base.—Stratford (Ia.) *Courier.*

*Finishing Amalgam Filling.* In finishing amalgam fillings, some teachers recommend drying off to a hard surface by pressure, with either bibulous paper or tin foil. Both of these practices, to my mind, should be strongly condemned, for the reason that the excluded mercury contains a certain proportion of the combined metals, and also because, when tin foil is employed, the correct proportions of silver and tin are altered so as to result in expansion or contraction of the malgam. My practice, therefore, is to have my assistant mix a certain proportion of the amalgam to almost a powder, which I work into the filling when nearly completed, thereby securing a hard filling with a more equable proportion to the constituents of the amalgam. I believe also in making, in nearly all cases, the amalgam fillings practically into inlays by burnishing the amalgam into the moist cement, thereby producing a much better filling.—W. N. Short, in *Commonwealth Dental Review.*

*Value of Prophylaxis.* If each patient desiring dental service, whether for the relief of immediate pain or the restoration of some tissue that has been lost through disease, should receive at the hands of his dentist proper prophylactic treatment, who can compute the resultant alleviation of suffering to mankind? The initial gingival infection is the forerunner of most of the destructive processes that result in the final exfoliation of the teeth. The whole alimentary tract, including the human mouth, is a veritable incubator for the propagation of germ life. Debris, lying in contiguous relation to tooth and gum, finds in the gingival trough a place of lodgment. The infection of this debris soon destroys the epithelial layer of the mucosa lying next to the tooth, opening a way for the passage of bacteria into the vessels, and thence into the blood stream. It is here that we may observe a focus of infection.—Paul R. Stillman, in *The Dental Cosmos.*

*Concerning Pulp Devitalization.* Dr. Henry Ulrich has said that out of one thousand devitalized teeth examined by him, over seventy per cent. showed well-defined abscesses. Other investigators have shown even higher percentages, as high as eighty-five per cent. from cultures made from blind abscesses. Such an appalling percentage of failures ought to keep every thinking man from practicing pulp devitalization and root filling except it be under the most favorable circumstances and conditions, and then only with the full knowledge and consent of the patient who has been advised of the possibilities that may occur.—W. L. Stamper, in *The Dental Summary.*

*Another Hygienist School.* The Forsyth Dental Infirmary has added a training school for Oral Hygienists. The Massachusetts law authorizes the employment of dental hygienists in public institutions. The course of instruction will cover twelve months. The entrance requirements is a high school education, and the candidate must be eighteen years of age. A course of dental training will also be offered to medical graduate nurses.

*A Worm Turns.* I hereby give notice to the doctors of Canton that from this date my price to them will be fifty cents for each shave and one dollar for each haircut, and that double prices will be charged for all work done after seven o'clock in the evening, and if it shall be necessary for me to visit them and do work for them while confined to their home by sickness an additional charge of twenty-five cents per foot for every foot traversed going and coming will be made.—E. P. Arquitt, in The Canton (N. Y.) *Plaindealer.*

